# Prevalence and associated risk factors of undercorrected refractive errors among people with diabetes in Shanghai

**DOI:** 10.1186/s12886-017-0620-2

**Published:** 2017-11-28

**Authors:** Mengjun Zhu, Xiaowei Tong, Rong Zhao, Xiangui He, Huijuan Zhao, Jianfeng Zhu

**Affiliations:** 1 Shanghai Eye Disease Prevention and Treatment Center, No. 380 Kangding Road, Jingan District, Shanghai, 200040 China; 2 Shanghai Hospital Development Center, Shanghai, 200040 China; 3 Center of Disease Control and Prevention of Baoshan District, Shanghai, 201901 China

**Keywords:** Undercorrected refractive error, Diabetes, Visual impairment, Causes, China

## Abstract

**Background:**

To investigate the prevalence and risk factors of undercorrected refractive error (URE) among people with diabetes in the Baoshan District of Shanghai, where data for undercorrected refractive error are limited.

**Methods:**

The study was a population-based survey of 649 persons (aged 60 years or older) with diabetes in Baoshan, Shanghai in 2009. One copy of the questionnaire was completed for each subject. Examinations included a standardized refraction and measurement of presenting and best-corrected visual acuity (BCVA), tonometry, slit lamp biomicroscopy, and fundus photography.

**Results:**

The calculated age-standardized prevalence rate of URE was 16.63% (95% confidence interval [CI] 13.76–19.49). For visual impairment subjects (presenting vision worse than 20/40 in the better eye), the prevalence of URE was up to 61.11%, and 75.93% of subjects could achieve visual acuity improvement by at least one line using appropriate spectacles. Under multiple logistic regression analysis, older age, female gender, non-farmer, increasing degree of myopia, lens opacities status, diabetic retinopathy (DR), body mass index (BMI) index lower than normal, and poor glycaemic control were associated with higher URE levels. Wearing distance eyeglasses was a protective factor for URE.

**Conclusion:**

The undercorrected refractive error in diabetic adults was high in Shanghai. Health education and regular refractive assessment are needed for diabetic adults. Persons with diabetes should be more aware that poor vision is often correctable, especially for those with risk factors.

## Background

According to recent estimates by the International Diabetes Federation, the worldwide diabetes prevalence has reached 8.3%, and approximately 382 million adults have had diabetes [1]. In China, the recent data show that the prevalence of diabetes in adults aged 20 years or older has increased to an alarming 11.6% [2]. The population size of Chinese patients with diabetes is estimated to be 92.4–98.4 million, and the incidence of diabetes has strongly increased [1, 3]. Persons with diabetes are more likely to be visually impaired [4] and may have ocular complications, such as diabetic retinopathy (DR), which is the leading cause of visual impairment (VI). As a result, for a long time, the public health strategies for diabetic people in China were early detection and treatment of the ocular complications of diabetes. Recently, a study indicated that nearly two-thirds of adults with diabetes who had visual impairment could correct their vision with an accurate corrective prescription for glasses or contact lenses [4]. To date, data regarding the prevalence of undercorrected refractive errors in the diabetic population in China are scarce. Shanghai is an ageing society, and the use of the elderly as our study population was motivated by several additional considerations. First, the prevalence of URE is relatively high in the elder population [4]. Second, most people ≥60 y in Shanghai have retired, and their enumeration can be more easily obtained from the official resident register than for younger people. The elderly are more prone to falls, and low vision has been documented to increase this risk [5, 6]; thus, visual correction is helpful for reducing the risk of injury and improving quality of life [7]. Therefore, the present study aimed to estimate the prevalence and associated risk factors for undercorrected refractive errors in people with diabetes. These data will be essential to the guidance and evaluation of public health policies designed to reduce the burden of visual impairment among elderly persons with diabetes in China.

## Methods

### Study population

The study subjects were recruited from the Baoshan Eye Study, which was a community-based, cross-sectional study of vision and eye diseases among subjects 60 years of age and above in Dachang, Shanghai, China. Baoshan District, with a population of 864,346 as of December 31, 2009, is located in the north of Shanghai. This area is representative of regions that are experiencing progressive urbanization as a result of China’s rapid economic growth. The study design and research methodology have been described in detail previously [8]. Based on previous studies, the prevalence of URE was anticipated to be 21.7% [9], and the allowable error bound to be 20% with 95% confidence levels. An 85% response rate and a design effect of 1.5 were used to calculate the requisite sample size of 611 (according to the formula N = Z^2^(p)(1-p)/B^2^ (*p* = 0.217, B = 0.217 × 0.20, Z = 1.96)). Overall, 743 persons who were identified as diabetic according to the registered medical records of the residents’ committee and community hospitals were eligible for the study. Vacant households, residents who died before being contacted, inpatients, subjects who refused to participate in the examination, and those who suffered from deafness or mental retardation were excluded. Finally, 680 subjects participated in this study. The current investigation followed the tenets of the Declaration of Helsinki and was approved by the Ethical Committee of the Shanghai Eye Disease Prevention and Treatment Center. All participants signed written informed consent before participating in the study.

### Enumeration and examination procedures

The study consisted of a structured questionnaire used to obtain baseline information on demographic data (age, gender, occupation, insurance, and education level), personal medical history (diabetes mellitus, hypertension, hyperlipidaemia, cardiovascular disease previously diagnosed by a physician, and duration and treatment of corresponding disease), family history of eye diseases, wearing and availability of glasses, and lifestyle (smoking and alcohol intake).

Standardized ophthalmic examinations beginning with a visual acuity (VA) test were performed by ophthalmologists, optometrists, and technicians. Presenting VA (with spectacles if worn) and VA after refractive correction were measured using a logMAR chart with tumbling E at a distance of 4 m. Autorefraction (Topcon KR-8900, Japan) was performed for all subjects independent of the VA. A subjective refraction exam was performed only for those with VA worse than 16/20. Slit lamp examination, tonometry, and a dilated biomicroscopic fundus examination were performed by ophthalmologists. Digital monoscopic photographs of the optic disc and macula were obtained (Canon CR6–45 nm, Japan). All fieldwork was conducted from October 2009 to December 2009.

In the overall study group (*n* = 680), refraction was not performed on 2 subjects (due to the blind eye), and we referred to their former refractive record. Thus, refraction data were available for all 680 subjects. The fundus could not be observed clearly in 10 subjects because of the effect of lens opacities. Hence, if DR or other fundus diseases had been diagnosed by a physician previously, we recorded yes. If no relevant diagnosis had been made before, we recorded no. We also excluded 31 (12.9%) subjects who had a history of cataract surgery and were either pseudophakic or aphakic. Finally, 649 subjects were included in this study. All subjects underwent measurement of systolic and diastolic blood pressure (mmHg), height, and weight. We also recorded the glycosylated haemoglobin of all subjects, which was examined by community hospitals within 1 week of study commencement. Fasting blood sugar estimation was not performed in this study.

### Definitions

Undercorrected refractive error was defined as a presenting visual acuity in the better eye worse than 6/12, with an improvement of at least 2 lines in BCVA compared to the presenting visual acuity [9]. The World Health Organization categories of vision loss were used to define blindness and severe visual impairment [10]. For mild and moderate visual impairment, a similar definition was used, which has been published in previous studies [11, 12]. Blindness was defined as a VA (with glasses for distance if normally worn or unaided if glasses for distance not worn) of <3/60 in the better eye. Severe visual impairment (SVI) was defined as a VA of <6/60 to 3/60 in the better eye. Moderate visual impairment (Mod VI) was defined as a VA of <6/18 to 6/60 in the better eye.

Mild visual impairment (Mild VI) was defined as a VA of <6/12 to 6/18 in the better eye. Emmetropia was defined as a spherical equivalent between −0.50 D and +0.50 D [12]. Myopia was defined as a spherical equivalent of less than −0.50 D, and hyperopia was defined as a spherical equivalent of greater than +0.50 D. For further analysis, myopia was classified into the following three groups: mild (−0.5 to −3.0 D), moderate (> − 3.0 to −6.0 D), and severe (> − 6.0 D). Grading of lens opacities was performed using the Lens Opacities Classification System (LOCS) III (LOCS chart III, Leo T. Chylack, Harvard Medical School, Boston, MA). Significant cortical and posterior subcapsular opacification was defined as greater than grade 2, and significant nuclear opacification was defined as greater than grade 4. DR was diagnosed according to the Early Treatment of Diabetic Retinopathy Study (ETDRS) criteria [13]. Other retinal diseases included optic atrophy, diabetic macular oedema, senile macular degeneration, high myopia macular degeneration, vascular retinopathy, or retinal detachment. If subjects had a combination of any one of the above diseases, we recorded the data as yes. Glycaemic control was categorized as normal (glycosylated haemoglobin [HbA1c] < 5.6), good (HbA1c 5.6–7.0), fair (HbA1c 7.1–8.0), unsatisfactory (HbA1c 8.1–10.0), or poor (HbA1c > 10) [14]. Individuals were classified as lean (male, <20; female, <19), normal (male, 20–25; female, 19–24), overweight (male, 25–30; female, 24–29), or obese (male, >30; female, >29) according to the calculated BMI [15]. Educational status was classified as illiterate, primary school (1–9 years), or secondary school or higher (>9 years).

### Statistical analysis

Odds ratios (ORs) and 95% confidence intervals (CIs) were calculated. Proportions were compared using the Chi-square test, and the means were compared using the *t*-test if parametric assumptions were fulfilled. Univariate analysis was used to examine whether there was a statistically significant association between each of the independent variables and URE. *P* < 0.05 was considered statistically significant. To determine whether independent variables were predictive factors for URE, multiple logistic regression analysis was used to fit the best model for independent variables (all the variables analysed in the univariate analysis were included in multivariate models), with URE as the dependent variable. Population prevalence rates for URE were also calculated by direct age standardization to the 2000 Shanghai population census.

## Results

In the study, 743 persons with diabetes aged ≥60 years were enumerated, and 680 (response rates: 91.52%; 95% CI: 89.52%–93.52%) were examined. Of the 680 subjects, 31 (4.56%) subjects had a history of cataract surgery and were either pseudophakic or aphakic. Because we mainly discussed URE, these participants were excluded. Therefore, data from 649 subjects were analysed. A comparison of demographic information and some of the variables in the subjects who were excluded or included in the study revealed that the subjects excluded from the study had a longer duration of diabetes than those included in the study (10.35 years vs. 7.57 years, *P* = 0.018). There were no significant differences in the other variables between the two groups (Table 1).

**Table 1:** Comparison of subjects included in and excluded from this study

	Excluded (*n* = 31)	Included (*n* = 649)	*P* ^a^
Age (mean ± SD)			
60–69	62.00 ± 1.60	62.87 ± 2.86	0.176
70–79	74.88 ± 2.42	74.23 ± 2.64	0.332
80~	84.50 ± 3.83	84.26 ± 5.32	0.915
Gender			0.060
Male	22.58%	40.52%	
Female	77.42%	59.48%	
Insurance status			0.755
No	0	0.92%	
Yes	100%	99.08%	
Occupation			0.206
Farmer	9.68%	17.10%	
Non-farmer	90.32%	82.90%	
Level of education			0.106
Illiteracy	22.58%	13.10%	
Primary school	54.84%	47.46%	
Secondary school or higher	22.58%	39.45%	
Hypertension	61.29%	62.25%	0.527
Hyperlipidaemia	3.23%	2.77%	0.593
Smoker	12.90%	18.18%	0.319
Alcohol use	9.68%	1.85%	0.200
BMI (mean ± SD)	24.58 ± 3.66	24.58 ± 3.43	0.999
Duration of diabetes (y) (mean ± SD)	10.35 ± 6.98	7.57 ± 6.36	0.018*
HbA1c (g%) (mean ± SD)	7.97 ± 3.31	7.58 ± 2.19	0.355
Spherical equivalent refractive error (SER) (mean ± SD)	−0.95 ± 1.83	−0.88 ± 3.07	0.843
Diabetic retinopathy	9.68%	4.62%	0.186

^a^Chi-square test was used for gender, insurance status, occupation, level of education, hypertension, hyperlipidaemia, smoking status, alcohol use, and diabetic retinopathy. Unpaired t-test was used for other variables

*P < 0.05

**Fig. 1:**
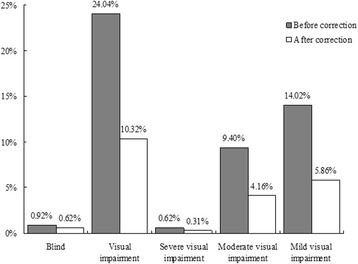
Prevalence of visual impairment before and after correction with appropriate spectacles

Of the 649 subjects, 6 subjects were presenting blindness, and 156 subjects were presenting VI (VA of less than 6/12) in the better eye. The prevalence of mild, moderate, and severe VI was 14.02%, 9.40%, and 0.62%, respectively, and was 5.86%, 4.16%, and 0.31%, respectively, with refractive correction. After appropriate correction, only 4 subjects were blind, and 67 subjects still had VI. Notably, more than half of the visual impairment subjects (89/156) could be corrected by wearing prescription spectacles (Fig. 1).

The prevalence rate of URE in the diabetic population is summarized in Table 2. Of the 649 subjects in our study, 99 (15.25%) had URE. The calculated age-standardized prevalence rate of URE was 16.63% (95% CI: 13.76–19.49). The prevalence of URE increased with age (*P* < 0.001). The prevalence was 9.20% in the youngest age group (60–69 years) and up to 32.89% in the oldest age group (≥80 years). URE was also more common in women than in men (*P* = 0.025). The rate of URE among women was 17.88%, and the value was 11.41% in men. Significant age-associated differences were noted in both women and men.

**Table 2:** Prevalence rates of undercorrected refractive error

	With glasses *n* (%)				Without glasses *n* (%)				Total *n* (%)			
	*N*	*n*	Prevalence Rate % (95% CI)	*P*	*N*	*n*	Prevalence Rate % (95% CI)	*P*	*N*	*n*	Prevalence Rate % (95% CI)	*P*
All (y)				0.044				<0.001				<0.001
60–69	82	10	12.20 (5.11–19.28)		320	27	8.44 (5.39–11.48)		402	37	9.20 (6.38–12.03)	
70–79	27	5	18.52 (3.87–33.17)	0.408	144	32	22.22 (15.43–29.01)	<0.001	171	37	21.64 (15.47–27.81)	<0.001
80~	15	5	33.33 (9.48–57.19)	0.037	61	20	32.79 (21.01–44.57)	<0.001	76	25	32.89 (23.54–44.88)	<0.001
Total	124	20	16.13 (9.66–22.60)		525	79	15.05 (11.99–18.11)		649	99	15.25 (12.49–18.02)	
Men (y)				0.054				0.011				0.001
60–69	40	3	7.50 (−0.66–15.66)		128	8	6.25 (2.06–10.44)		168	11	6.55 (2.81–10.29)	
70–79	18	2	11.11 (−3.41–25.63)	0.650	50	11	22.00 (10.52–33.48)	0.002	68	13	19.12 (9.77–28.46)	0.004
80~	9	3	33.33 (2.53–64.13)	0.030	18	3	16.67 (−0.55–33.88)	0.140	27	6	22.22 (6.54–37.90)	0.007
Subtotal	67	8	11.94 (4.18–19.70)		196	22	11.22 (6.81–15.64)		263	30	11.41 (7.56–15.25)	
Women (y)				0.212				<0.001				<0.001
60–69	42	7	16.67 (5.40–27.94)		192	19	9.90 (5.67–14.12)		234	26	11.11 (7.08–15.14)	
70–79	9	3	33.33 (2.53–64.13)	0.253	94	21	22.34 (13.92–30.76)	0.004	103	24	23.30 (15.14–31.47)	0.004
80~	6	2	33.33 (−4.39–71.05)	0.328	43	17	39.53 (24.92–54.15)	<0.001	49	19	38.78 (25.13–52.42)	<0.001
Subtotal	57	12	21.05 (10.47–31.64)		329	57	17.33 (13.24–21.41)		386	69	17.88 (14.05–21.70)	
P(sex)			0.171				0.059				0.025	

Among 525 examined subjects who did not have spectacle correction, the prevalence of URE was 15.05%, which increased from 8.44% among those 60 to 69 years old to 34.43% among subjects greater than 80 years old (*P* < 0.001). Age-associated differences were also observed in women and men.

Among 124 subjects who habitually wore spectacles, the prevalence of URE among 60–69 year olds, 70–79 year olds, and greater than 80 year olds was 12.20%, 18.52%, and 33.33%, respectively (*P* = 0.044). No significant age or sex-associated differences were noted.

Table 3 shows the improvement in the participants’ vision achieved by correcting their uncorrected or miscorrected refractive errors. Using appropriate spectacles improved the VA by at least one line in 44.84% (291/649) of the studied population and four lines or more in 5.55% (36/649). These figures were more pronounced among participants with VI. Among 71 participants whose VA was worse than 6/18 in the better eye, 43 (60.56%) had URE. In total, 49 subjects (69.01%) obtained an improvement of at least one line after refractive correction, and 26 (36.62%) experienced a four-line improvement in vision by wearing proper spectacles. The presenting VA was worse than 20/20 in the better eye of 518 participants, among whom 7.35% gained four lines of visual acuity with accurately prescribed spectacles. According to the definition, the prevalence of URE in subjects whose presenting VA was worse than 6/18, 20/40, and 20/20 in the better eye was 60.56%, 61.11%, and 28.43%, respectively.

**Table 3:** Visual improvement after correcting refractive errors

	Gained lines	Without glasses *n* (%)	With glasses *n* (%)	Total *n* (%)
Presenting vision worse than 6/18 in the better eye (*n* = 71)	0 lines	14 (28.57%)	8 (36.36%)	22 (30.99%)
	≥1 line	35 (71.43%)	14 (63.64%)	49 (69.01%)
	≥2 lines	30 (61.22%)	13 (59.09%)	43 (60.56%)
	≥3 lines	26 (53.06%)	9 (40.91%)	35 (49.30%)
	≥4 lines	23 (46.94%)	3 (13.64%)	26 (36.62%)
Presenting vision worse than 20/40 in the better eye (*n* = 162)	0 lines	28 (22.22%)	11 (30.56%)	39 (24.07%)
	≥1 line	98 (77.78%)	25 (69.44%)	123 (75.93%)
	≥2 lines	79 (62.70%)	20 (55.56%)	99 (61.11%)
	≥3 lines	50 (39.68%)	10 (27.78%)	60 (37.04%)
	≥4 lines	33 (26.19%)	3 (8.33%)	36 (22.22%)
Presenting vision worse than 20/20 in the better eye (*n* = 518)	0 lines	178 (43.31%)	48 (44.86%)	226 (46.57%)
	≥1 line	233 (56.69%)	59 (55.14%)	292 (56.37%)
	≥2 lines	119 (28.95%)	34 (31.78%)	153 (28.43%)
	≥3 lines	59 (14.36%)	14 (13.08%)	73 (14.22%)
	≥4 lines	33 (8.03%)	3 (2.80%)	36 (7.35%)

In 124 subjects who wore spectacles for distance vision, nearly half (59/124) achieved visual acuity improvement by at least one line using appropriate spectacles; however, the prevalence of URE was still 16.13% (20/124) in subjects with glasses. Only 48 (38.71%) subjects gained no benefit from a distance correction. In 525 subjects without spectacle correction, 233 (44.38%) obtained an improvement of at least one line after refractive correction, and 33 (6.29%) experienced a four-line improvement in vision by wearing proper spectacles. The prevalence of URE was 22.67% (119/525) in subjects without glasses.

Table 4 shows the association between URE and the various clinical and biochemical variables. URE was significantly associated with older age (70–79 years: OR: 2.72, 95% CI: 1.66–4.48; 80~ years: OR: 4.84, 95% CI: 2.69–8.69), female gender (OR: 1.69, 95% CI: 1.07–2.68), and myopia refractive error. A higher degree of myopia led to a higher risk for URE (low myopia: OR: 2.72, 95% CI: 1.34–5.50; moderate myopia: OR: 4.60, 95% CI: 1.97–10.78; high myopia: OR: 7.66, 95% CI: 3.29–17.84). Of the diabetes-related variables, lens opacities status (OR: 3.81, 95% CI: 2.22–6.51), diabetic retinopathy (OR: 2.52, 95% CI: 1.12–5.68), combined with other retinal diseases (OR: 1.98, 95% CI: 1.02–3.86), BMI index lower than normal (OR: 2.45, 95% CI: 1.01–5.92), and a high glycated haemoglobin concentration (≥10.0, OR: 4.41, 95% CI: 1.77–11.02) were risk factors for URE.

**Table 4:** Univariate and multivariate analysis of undercorrected refractive error in participants with Diabetes mellitus (*n* = 649)

	*N*	*n*	% (95% CI)	Univariate Odds Ratio (95% CI)	*P*	Multivariate Odds Ratio (95% CI)	*P*
Age, y					<0.001		<0.001
60–69	402	37	9.20 (6.38–12.03)	1		1	
70–79	171	37	21.64 (15.47–27.81)	2.72 (1.66–4.48)^‡^	<0.001	3.28 (1.78–6.07)^‡^	<0.001
80~	76	25	32.89 (22.33–43.46)	4.84 (2.69–8.69)^‡^	<0.001	3.93 (1.85–8.35)^‡^	<0.001
Total	649	99	15.25 (12.49–18.02)				
Age adjusted†			16.63 (13.76–19.49)				
Gender					0.026		0.044
Male	263	30	11.41 (7.56–15.25)	1		1	
Female	386	69	17.88 (14.05–21.70)	1.69 (1.07–2.68)^*^		1.79 (1.02–3.15)^*^	
Occupation					0.156		0.049
Farmer	111	18	16.22 (9.36–23.07)	1		1	
Non-farmer	538	135	25.09 (21.43–28.76)	1.59 (0.84–3.02)		2.17 (1.01–4.68)^*^	
Level of education					0.083		0.226
Illiteracy	85	15	17.65 (9.54–25.75)	1		1	
Primary school	308	55	17.86 (13.58–22.13)	1.01 (0.54–1.90)	0.964	1.79 (0.80–4.01)	0.156
Secondary school or higher	256	29	11.33 (7.45–15.21)	0.60 (0.30–1.18)	0.135	1.25 (0.50–3.09)	0.637
Insurance status					0.923		0.779
No	6	1	16.67 (−13.15–46.49)	1		1	
Yes	643	98	15.24 (12.46–18.02)	0.90 (0.10–7.78)		0.72 (0.05–10.05)	
Refractive error					<0.0001		<0.001
Emmetropia	156	12	7.69 (3.51–11.87)	1		1	
Hyperopia	228	25	10.96 (6.91–15.02)	1.50 (0.73–3.08)	0.271	1.62 (0.73–3.59)	0.234
Low myopia	168	31	18.45 (12.59–24.32)	2.72 (1.34–5.50)^†^	0.005	2.99 (1.36–6.55)^†^	0.006
Moderate myopia	53	14	24.62 (14.55–38.28)	4.60 (1.97–10.78)^†^	0.001	11.45 (3.86–33.92)^‡^	<0.001
High myopia	44	17	38.64 (24.25–53.02)	7.66 (3.29–17.84)^‡^	<0.0001	18.91 (5.97–59.92)^‡^	<0.001
Lens status					<0.0001		0.001
Normal	270	18	6.67 (3.69–9.64)	1		1	
Opacities	379	81	21.37 (17.24–25.50)	3.81 (2.22–6.51)^‡^		2.83 (1.50–5.36)^†^	
Diabetic retinopathy					0.026		0.035
No	619	90	14.54 (11.76–17.32)	1		1	
Yes	30	9	30.00 (13.60–46.40)	2.52 (1.12–5.68)^*^		2.99 (1.08–8.30)^*^	
Combined with other retinal diseases					0.045		0.995
No	597	86	14.41 (11.59–17.22)	1		1	
Yes	52	13	25.00 (13.23–36.77)	1.98 (1.02–3.86)^*^		1.00 (0.40–2.48)	
Duration of diabetes (y)					0.279		0.223
< 5	303	52	17.16 (12.92–21.41)	1		1	
5–10	212	24	11.32 (7.06–15.59)	0.62 (0.37–1.04)	0.068	0.60 (0.33–1.10)	0.100
10–15	80	13	16.25 (8.17–24.33)	0.94 (0.48–1.82)	0.847	0.56(0.25–1.28)	0.172
≥ 15	54	10	18.52 (8.16–28.88)	1.10 (0.52–2.32)	0.808	0.50 (0.19–1.27)	0.145
Drug use for diabetes					0.279		0.401
No	89	17	19.10 (10.93–27.27)	1		1	
Yes	560	82	14.64 (11.71–17.57)	0.73 (0.41–1.30)		0.73 (0.36–1.51)	
History of diabetic retinopathy therapy					0.053		0.205
No	630	93	14.76 (11.99–17.53)	1		1	
Yes	19	6	31.58 (10.68–52.48)	2.67 (1.00–7.19)		2.36 (0.63–8.90)	
HbA1c (g%)					<0.001		0.003
< 5.6	70	7	10.00 (2.97–17.03)	1		1	
5.6–7.0	260	31	11.92 (7.98–15.86)	1.22 (0.51–2.90)	0.655	0.74 (0.28–1.96)	0.546
7.1–8.0	138	18	13.04 (7.42–18.66)	1.35 (0.54–3.40)	0.525	0.85 (0.30–2.43)	0.765
8.1–10.0	105	18	17.14 (9.93–24.35)	1.86 (0.73–4.73)	0.191	1.21 (0.43–3.41)	0.721
≥ 10.0	76	25	32.89 (22.33–43.46)	4.41 (1.77–11.02)^†^	0.001	3.18 (1.11–9.12)^*^	0.031
BMI(kg/m^2^)					0.211		0.096
Normal	299	42	14.05 (10.11–17.99)	1		1	
Lean	28	8	28.57 (11.84–45.30)	2.45 (1.01–5.92)^*^	0.047	3.44 (1.19–9.91)^*^	0.022
Overweight	268	39	14.55 (10.33–18.77)	1.04 (0.65–1.67)	0.864	1.07 (0.61–1.90)	0.810
Obese	54	10	18.52 (8.16–28.88)	1.39 (0.65–2.97)	0.395	1.74 (0.69–4.39)	0.244
Hypertension					0.325		0.894
No	245	33	13.47 (9.19–17.74)	1		1	
Yes	404	66	16.34 (12.73–19.94)	1.25 (0.80–1.97)		0.96 (0.55–1.69)	
Hyperlipidaemia					0.620		0.577
No	631	97	15.37 (12.56–18.19)	1		1	
Yes	18	2	11.11 (−3.41–25.63)	0.69 (0.16–3.04)		0.61 (0.11–3.42)	
Smoker					0.780		0.529
No	531	82	15.44 (12.37–18.52)	1		1	
Yes	118	17	14.41 (8.07–20.74)	0.92 (0.52–1.62)		0.67 (0.19–2.37)	
Alcohol use					0.791		0.715
No	537	81	15.08 (12.06–18.11)	1		1	
Yes	112	18	16.07 (9.27–22.87)	1.08 (0.62–1.88)		1.26 (0.36–4.38)	
Cardiovascular disease					0.487		0.832
No	595	89	14.96 (12.09–17.82)	1		1	
Yes	54	10	18.52 (8.16–28.88)	1.29 (0.63–2.66)		1.10 (0.44–2.74)	
Distance eyeglasses wearing					0.703		0.011
No	525	79	15.05 (11.99–18.11)	1		1	
Yes	124	20	16.13 (9.66–22.60)	1.09 (0.64–1.85)		0.34 (0.15–0.78)^*^	

*P < 0.05

†*P* < 0.01

‡*P* < 0.001

In the final multiple logistic regression analysis controlling for all covariates, older age, female gender, non-farmer, myopia refractive error, lens opacities status, diabetic retinopathy, BMI index lower than normal, and poor glycaemic control were associated with higher levels of URE. In contrast, wearing distance eyeglasses during the eye examination was a protective factor for URE. Other variables, such as level of education, insurance status, and duration of diabetes were not significantly associated with URE.

## Discussion

Visual Impairment and diabetes are both very common in the older population [2, 3, 11]. In response to the global initiative for elimination of avoidable blindness raised by the WHO, Vision 2020, it is necessary to understand the major causes of VI in this special group and how to avoid these risks. As a survey of URE in the elderly diabetic population, our study showed that if refractive correction was available, the prevalence of mild, moderate, and severe VI could be reduced from 14.02%, 9.40%, and 0.62%, respectively, to 5.86%, 4.61%, and 0.31%, respectively. Our study also found that for diabetic subjects with presenting vision worse than 6/18, the occurrence of URE was 60.56%, and more than one-third of them (36.62%) gained four or more lines of visual improvement. For presenting vision worse than 20/40, these two indicators were 76.1% and 62.0%, respectively. Hence, these results indicated that a significant proportion of visual loss was due to inadequately corrected refractive error.

To the best of our knowledge, this is the first study to document URE among senior individuals with diabetes in urban China, and the rate of URE was 16.63%. The prevalence in the present study was somewhat higher than in Hong Kong [4]. Other studies on general populations have reported various rates. In a review, the rate of URE ranged from 9.55% to 21.7% [9, 15–18] (Table 5). Although the age of recruitment, the definition for URE, the sampling strategies, the geography, and the race were different from those in the present study, a similar phenomenon was noted. URE was the major cause of presenting blindness and VI both in general populations and among diabetic people in China.

**Table 5:** Comparison of prevalence and risk factors of undercorrected refractive error between the present study and various published studies

Study	Sample Size	Population	Age	Undercorrection in Study Population (%)	Undercorrection among Participants with Refractive Error (%)	Definitions	Multivariate Risk Factors
Present study	649	Diabetes Chinese	Mean: 68.37 ± 8.22 Range: 60–100	16.63%	10.96% of hyperopia subjects 23.40% of myopic subjects	Presenting visual acuity <20/40(Best-corrected VA -Presenting VA) ≥2 lines improvement	Older age, female gender, non-farmer, myopia, lens opacities status, DR, lower BMI, poor glycaemic control, not wearing spectacles
Hong Kong Correctable Visual Impairment Study [4]	2301	Type 2 diabetes mellitus	Mean: 61.4 ± 10.5 Range: 23–92	7.30%	NA	Presenting VA <6/18 that improved to no impairment (≥6/18) after refractive correction	Older age, more advanced stage of DR
Tanjong Pagar Survey [9]	1152	General Singaporean Chinese	40–79	21.7%	NA	Presenting VA <20/40 in the better eye (Best-corrected VA- Presenting VA) ≥2 lines improvement	Older age, lower education level, not wearing spectacles, cataracts
SiMES [15]	3115	General Singaporean Malays	Mean: 58 ± 11 Range: 40–80	20.4%	28.7% of hyperopia subjects 28.9% of myopic subjects	Presenting VA <20/40 in the better eye (Best-corrected VA- Presenting VA) ≥2 lines improvement	Older age, female sex, lower education level
Shihpai Eye Study [16]	1361	General Chinese	Mean: 72.2 Range: ≥65	9.55%	11.3% of hyperopic subjects 34.4% of myopic subjects	Presenting VA < 20/40 in the better eye (Best-corrected VA -Presenting VA) ≥2 lines improvement in better eye	Older age, non-emmetropic eye, not wearing spectacles and lower level of education
LALES [17]	6129	General Latinos	Mean: 54.9 ± 10.8 Range: ≥40	15.1%	NA	(Best-corrected VA-presenting VA) ≥2 lines improvement in better eye	Older age, lack of health insurance, lower education level, lower BMI, being unemployed
BMES [18]	3654	General Caucasians	Mean: 66.2 Range: 49–97	10.2%	53.9% of hyperopic subjects 12.7% of myopic subjects	Presenting VA <20/40 (Best-corrected VA - presenting VA) improvement of ≥2 lines in the better eye	Older age, living alone, occupations of trade and labourer, receipt of a government pension, hyperopia and duration from the last eye examination

*NA* not available

In our study, some risk factors for URE in the general population were also identified in the diabetic population, i.e., older age, female gender, non-farmer, myopic refractive error, and not wearing distance eyeglasses [9, 15–18]. Some risk factors that were shown in the general population, such as unemployment [17] and lower education level [15–17], were not observed in the present study among the diabetic population. Older age, as the most significant risk factor, was also observed in the Hong Kong study involving a population with diabetes [4]. In the general population, the probable explanation is that older subjects are more likely to have a lower level of education and lower economic status, which has been estimated to be associated with a higher prevalence of URE [15–17, 19, 20]. However, in the diabetic population, another primary cause of URE might be specific physical barriers caused by complications of diabetes, such as diabetic cardiovascular complications, diabetic peripheral neuropathy, and diabetic nephropathy, which have a higher incidence in the elderly. Females in our survey had a higher prevalence of URE than males. This disparity may be related to Chinese traditional culture. In China, females are given less attention than males in the family. Therefore, females have a lower educational level, leading to a lower socioeconomic status and reduced awareness of health care [8].

Our study shows that a higher degree of myopia is a significant risk factor for URE in diabetic people, with odds of 18.91 times for high myopia and 11.45 times for moderate myopia. In mild myopia, undercorrection would be helpful for near work among elderly subjects in daily life. However, in moderate and high myopia, the possible explanation for higher prevalence of URE is that they think the decrease in VA is due to the primary disease, i.e., diabetes. Second, the idea that undercorrection of myopia could inhibit myopia progression is prevalent among myopic persons.

In China, farmer is always a symbol of lower-income and lower education level, which makes people think farmer will be a risk factor of URE. However, farmer also means a healthy lifestyle in China. These individuals spend more time on outdoor activities and have less near distance work, which results in a lower prevalence of myopia in the farmer population [21]. As mentioned above, myopia was a significant risk factor of URE in the current analysis. Hence, farmers had a lower prevalence of URE in this survey.

We should pay more attention to a number of factors associated with diabetes, such as diabetic retinopathy, significant lens opacities, lower BMI, and worsening of glycaemic control (HbA1c (g%) ≥ 10.0). As is well known, DR is a major cause of blindness and VI in the diabetic population [22, 23]. However, in our survey, the prevalence of URE was shown to be higher in subjects with DR than in subjects without DR (30.00% vs. 14.54%), indicating that impaired vision could be improved even in individuals with DR. This finding could be explained by the fact that although DR can lead to a decrease in VA, this ocular complication is still primary in most subjects. URE is the leading cause of VI in individuals with DR. For example, in a study from Denmark, DR was found in 7% of diabetic patients and was not sight-threatening in any of the cases [24]. Individuals with DR often misunderstand that their vision loss is caused by ocular complications of diabetes and that nothing can be done to improve the situation, completely ignoring the possibility of URE.

In our study, persons with cataracts had nearly three-fold greater odds of having URE than persons without cataracts. In the general population, the view that cataract-induced vision loss can be corrected by surgery or appropriate refractive correction has been accepted by most people. However, almost all the health education for diabetes in Shanghai emphasizes the dangers of ocular complications in diabetes, which makes diabetic subjects think that their vision loss is inevitable and irreversible. In fact, a myopic shift can occur due to the expansion of the lens, and the vision loss caused by this period of cataracts may be completely corrected by spectacles.

Unexpectedly, in our study, lower BMI was found to be associated with a higher risk of URE. In the general population, lower BMI may represent a healthy lifestyle, healthy diet, and sufficient physical activities; however, in the diabetic population, a lower BMI may be seen as a sign of a catabolic state and perhaps be a precursor to death. Hence, a possible explanation is that lower BMI diabetic subjects have relatively serious physical complications that prevent them from attending regular healthcare visits and receiving optometric service. Another explanation is that compared with obese and overweight individuals, lower BMI subjects have less awareness of their medical problems and health issues.

Glycosylated haemoglobin is stable and is a reliable to reflect the average blood glucose levels nearly 8 to 12 weeks before detection; as such, it is the gold standard for monitoring diabetes. In our study, worsening of glycaemic control resulted in a higher risk of URE by nearly 3-fold because hyperglycaemia causes a transient myopic shift in poorly controlled diabetic subjects [14, 25, 26]. Our data suggest that adults with diabetes should pay greater attention to the control and systemic management of their diabetes and also visit the eye service and optometric service immediately when they are experiencing loss of vision to determine the cause and subsequent treatment to reduce the risk of URE.

These risk factors for URE, especially factors associated with diabetes, have important implications in planning public health strategies for urban China, where government health insurance is widely available. Risk factors associated with diabetes remind us that lack of knowledge and awareness of refractive error may be the primary cause of URE in the diabetic population. We recommend establishing comprehensive eye care services and improving health education programmes, especially optometric service and knowledge of URE in the diabetic population. Most eye screening programmes and health education for people with diabetes in Shanghai emphasize cataracts and DR, ignoring the importance of refractive error.

Our study showed that wearing spectacles is a protective factor against URE. A supply of suitable glasses is the most efficient and economical way to improve vision and reduce the prevalence of URE. We suggest improving the quality of optometric service and reducing the cost of purchasing spectacles to encourage the use of spectacles. Although the cost of refraction is already covered by the health insurance system in Shanghai, the cost of spectacles is still expensive, and this may be an important reason why refractory error remains uncorrected in some individuals.

This study has several limitations. First, this study was conducted only among the senior diabetes population aged 60 or above. However, diabetes is also a common metabolic disorder in adults aged 20–60 years, and it is meaningful to explore the prevalence of URE and associated risk factors in this age group. Studies including this age group are warranted. Second, the study was conducted in a single centre, which might not be similar to other geographic areas in Shanghai. Third, data were based on known diabetes only. Patients with undiagnosed diabetes might lead to a potential source of bias. Fourth, glycated haemoglobin values and type of diabetes were not available. Considering that there may be different risk factors between persons with type 1 diabetes and type 2 diabetes, additional studies may be needed to elucidate any differences.

## Conclusion

In conclusion, the present study found that approximately 16.63% of diabetic adults aged 60 years and above had URE. URE is also a significant cause of VI among diabetic adults. Apart from some risk factors of URE both in the general population and diabetic population, such as older age, female gender, non-farmer, myopic refractive error, and not wearing distance eyeglasses, we should pay more attention to the risk factors associated with diabetes, such as DR, significant lens opacities, lower BMI, and worsening of glycaemic control (HbA1c (g%) ≥ 10.0). Health education programmes should disseminate basic optometric knowledge and give correct guidance to the diabetic population. Regular screening, including a simple refractive assessment, is needed to reduce the magnitude of URE in subjects with diabetes. Persons with diabetes ought to be more aware that poor vision is often correctable.

## Abbreviations

BCVA: Best-corrected visual acuity
CI: Confidence interval
DR: Diabetic retinopathy
Mild VI: Mild visual impairment
Mod VI: Moderate visual impairment
OR: Odds ratio
SER: Spherical equivalent refractive error
SVI: Severe visual impairment
URE: Undercorrected refractive error
VA: Visual acuity
VI: Visual impairment
